# The Effect of rTMS over the Different Targets on Language Recovery in Stroke Patients with Global Aphasia: A Randomized Sham-Controlled Study

**DOI:** 10.1155/2019/4589056

**Published:** 2019-07-29

**Authors:** Caili Ren, Guofu Zhang, Xinlei Xu, Jianfeng Hao, Hui Fang, Ping Chen, Zhaohui Li, Yunyun Ji, Qingjie Cai, Fei Gao

**Affiliations:** ^1^Department of Neurological Rehabilitation, Wuxi Tongren Rehabilitation Hospital of Nanjing Medical University, Wuxi, Jiangsu Province, China; ^2^Department of Psychiatry, The Affiliated Wuxi Mental Health Center of Nanjing Medical University, Wuxi, Jiangsu Province, China

## Abstract

**Objective:**

To evaluate and compare the effects of repetitive transcranial magnetic stimulation (rTMS) over the right pars triangularis of the posterior inferior frontal gyrus (pIFG) and the right posterior superior temporal gyrus (pSMG) in global aphasia following subacute stroke.

**Methods:**

Fifty-four patients with subacute poststroke global aphasia were randomized to 15-day protocols of 20-minute inhibitory 1 Hz rTMS over either the right triangular part of the pIFG (the rTMS-b group) or the right pSTG (the rTMS-w group) or to sham stimulation, followed by 30 minutes of speech and language therapy. Language outcomes were assessed by aphasia quotient (AQ) scores obtained from the Chinese version of the Western Aphasia Battery (WAB) at baseline and immediately after 3 weeks (15 days) of experimental treatment.

**Results:**

Forty-five patients completed the entire study. The primary outcome measures include the changes in WAB-AQ score, spontaneous speech, auditory comprehension, and repetition. These measures indicated significant main effect between the baseline of the rTMS-w, rTMS-b, and sham groups and immediately after stimulation (*P*<0.05). Compared with the sham group, the increases were significant for auditory comprehension, repetition, and AQ in the rTMS-w group (*P*<0.05), whereas the changes in repetition, spontaneous speech, and AQ tended to be higher in the rTMS-b group (*P*<0.05).

**Conclusions:**

Inhibitory rTMS targeting the right pIFG and pSTG can be an effective treatment for subacute stroke patients with global aphasia. The effect of rTMS may depend on the stimulation site. Low-frequency rTMS inhibited the right pSTG and significantly improved language recovery in terms of auditory comprehension and repetition, whereas LF-rTMS inhibited the right pIFG, leading to apparent changes in spontaneous speech and repetition.

## 1. Introduction

Stroke-related aphasia is one of the most common consequences of cerebrovascular diseases and occurs in one-third of acute or subacute stroke patients [1]. Global aphasia is one of the most serious and common aphasia types in acute and subacute stroke patients. This type is usually caused by infarction of the left middle cerebral artery. Patients with global aphasia have difficulties with communication, which is affected gravely and comprehensively in the domains of spontaneous speech, auditory comprehension, naming, and repetition. The most important period of language recovery usually occurs in the first to the third month after stroke, which is the key time for neurophysiological restoration and reorganization of the language cortex [2].

Mounting studies have demonstrated that inhibitory low-frequency repetitive transcranial magnetic stimulation (LF-rTMS) (≤1 Hz) over the unaffected hemisphere can improve language function in poststroke aphasic patients with left-hemispheric lesions [3–6]. Language functional recovery occurs because of compensatory facilitation of the left hemisphere following the reduction of interhemispheric inhibition by suppressive LF-rTMS. Ideally, when used therapeutically for aphasia to reduce interhemispheric inhibition and facilitate the neural activity of the compensatory areas, LF-rTMS should be applied to an area homologous to the compensatory areas with impaired language function [7]. Our previous work [8] identified seven randomized controlled trials involving 160 stroke patients for a meta-analysis that investigated the positive effect of low-frequency rTMS targeting the pars triangularis of the right posterior inferior frontal gyrus (pIFG) [9–12]. Abo et al. [13] reported that low-frequency rTMS over the superior temporal gyrus (STG) of the temporal lobe improved language function in patients with fluent aphasia. A positron emission tomographic study found right hemisphere activation in STG in patients with Wernicke's aphasia [14]. The right posterior part of the superior temporal gyrus (pSTG), homotopic to the left Wernicke's area, is the other optimal target of rTMS stimulation.

The right pars triangularis (BA45) was selected as a stimulation site because previous imaging studies have shown that activation occurs in all patients in whom the perisylvian language cortex of the dominant hemisphere is damaged, and 1 Hz inhibitory TMS over the right pars triangularis but not the pars opercularis can improve anomia in patients with aphasia [15]. The rationale for the utilization of the right pSTG as the other stimulation site was that it is an important area of the language network and a key point of the ventral stream, which is involved in mapping sound onto meaning [16]. Therefore, this suggests that LF-rTMS over the right pSTG in global aphasia may improve auditory comprehension significantly by promoting a recovery semantic language network.

However, little is known about the effects of LF-rTMS over the right pSTG combined with speech and language therapy (SLT) in the treatment of subacute global aphasia poststroke. The present randomized sham-stimulation-controlled study aimed to investigate the efficacy of LF-rTMS over the right pSTG or the right pIFG in global aphasic patients with subacute stroke.

## 2. Materials and Methods

### 2.1. Subjects

A total of 45 right-handed (assessed by the Edinburgh Handedness Inventory; Oldfield, 1971) subjects suffering from subacute stroke with global aphasia participated in this experiment. All subjects were native Chinese speakers aged 45 to 75 years.  Table 1 provides the detailed demographic and clinical information of the participants. The inclusion criteria were as follows: (1) a first-ever left-sided middle cerebral artery (MCA) stroke with the lesion site verified by magnetic resonance imaging (MRI); (2) the time between 4 and 12 weeks after suffering from the stroke; (3) global aphasia defined by WAB-AQ scores; and (4) written informed consent from all subjects who participated in the study.

The exclusion criteria were as follows: (1) vision and hearing disabilities that might interfere with diagnostic and therapeutic treatment; (2) medications altering the level of cortical excitability (e.g., antiepileptics, neuroleptics or benzodiazepines); (3) a history of substance abuse, premorbid dementia or any neuropsychiatric diseases; and (4) contraindications for rTMS according to the safety guidelines [17, 18].

The Ethics Committee of the Medical University of Nanjing approved the study protocol. We registered the protocol of the present randomized controlled study in the Chinese Clinical Trial Registry (no. ChiCTR-IPR-15007382).

### 2.2. Procedure

This study was randomized, double-blinded, and sham controlled. A completely randomized digital table was used to generate the random allocation sequence. The patients were randomly assigned to three groups: those receiving real inhibiting rTMS on the right pars triangularis of the pIFG, which is the homolog of the left Broca's area (the rTMS-b group); those receiving real inhibiting rTMS on the right pSTG, which is the homolog of the left Wernicke's area (the rTMS-w group); and those receiving sham rTMS (the sham group), all in combination with SLT. The allocations were stored in sealed, numbered envelopes. The subjects did not know whether they were receiving real or sham rTMS. The language therapist assessed speech and language abilities and was blinded to the patients' group assignments. All subjects, investigators (except the investigator responsible for rTMS application), clinicians, speech, and language therapists were blinded to patient assignment to real or sham rTMS.

The therapeutic procedure consisted of rTMS sessions and SLT. Subjects in all three groups underwent SLT sessions for 30 minutes immediately after finishing rTMS treatment from Monday to Friday for 3 weeks. The speech and language training mainly focused on the comprehension and expression of spoken language. The rehabilitation program focused on specific training to stimulate various aspects of the language system (e.g., semantic, phonological, syntactic or motor).

A language assessment was performed at baseline and immediately after 3 weeks of real or sham rTMS treatment using the Western Aphasia Battery (WAB), which evaluated the capabilities of spontaneous speech, auditory comprehension, repetition and naming. The AQ was obtained after the examination. This measure reflects the severity of aphasia and can be used as an index for evaluating the improvement and deterioration of aphasia. The highest AQ score is 100, and the normal range is 98.4-99.6. AQ<93.8 is classified as aphasia. When the measure results of patients reach the score of spontaneous speech 0~4, auditory comprehension 0~3.9, repetition 0~4.9, and naming 0-6, they are defined as the patients of global aphasia.

### 2.3. Transcranial Magnetic Stimulation

rTMS was performed with a MagPro® (MagVenture Company, Farum, Denmark) equipped with an air-cooled figure-of-eight coil (each loop was 70 mm in diameter). The subjects were seated in a chair that allowed their head to lean on the headrest to ensure that it was immobile during the rTMS procedure. The coil was placed tangentially to the scalp over the right pIFG (F4 site on a standard EEG-10/20) or the pSTG (CP6 site on a standard EEG-10/20). Each LF-rTMS session consisted of 1,200 pulses and lasted 20 minutes. Magnetic stimulation was applied at 80% of the resting motor threshold (RMT) at a 1-Hz frequency. RMT was determined in each subject once before treatment and was defined as the minimum stimulus intensity able to elicit a motor evoked potential of at least 50 mV in 5 or more of 10 consecutive stimulations. MEP was recorded from the first dorsal interosseus muscle of the unaffected hand. The stimulation parameters were chosen according to current safety guidelines for rTMS [19]. The sham stimulation used the same coil and was placed vertically over the vertex with the same stimulation parameters used for the real rTMS procedure.

### 2.4. Sample Size Calculation

Based on previous studies [9, 20], we expected an effect size of 0.5, with an *α* of 0.05 and a power of 0.8. The minimum sample size in each group was n=14. If the dropout rate is less than 20%, we need at least 18 participants in each group.

### 2.5. Statistical Analysis

The necessary sample size was estimated by referring to the previous randomized controlled study [9]. The data analyses were performed with SPSS version 22.0 for Windows statistical software. Differences in categorical data were analyzed using the Chi-square test. Descriptive data were reported as the mean±SD for normally distributed data or as the median (interquartile range) for discrete variables of baseline characteristics and the language function scores of each group. A 2-factor repeated measures ANOVA was performed to test for differential treatment effects on WAB-AQ and WAB subtests. Post hoc analyses were applied to multiple comparisons. Differences were considered statistically significant when* P*<0.05.

## 3. Results

### 3.1. Participant Characteristics

**Figure 1:**
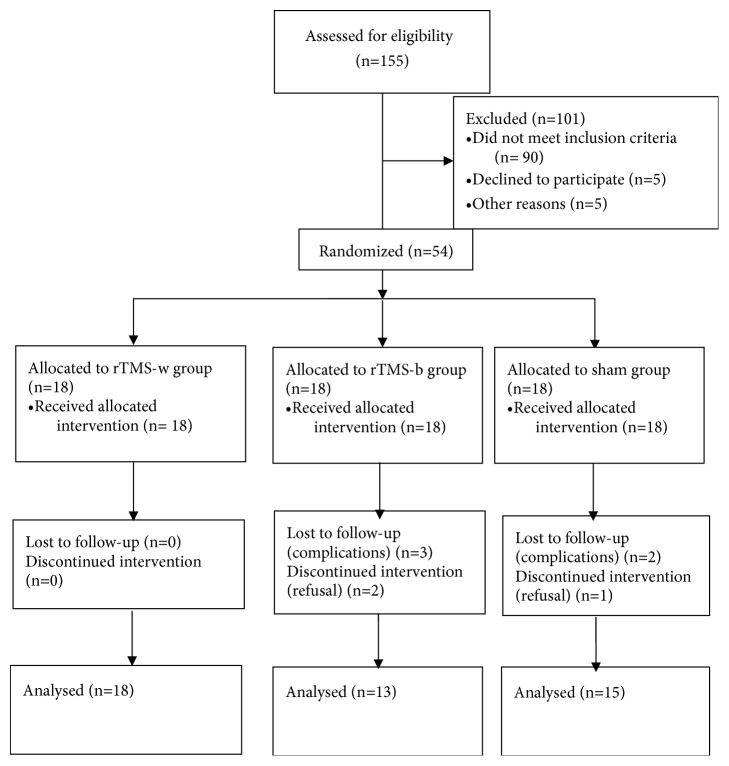
CONSORT diagram of patient flow throughout the study.

A total of 54 patients participated in the study based on the inclusion and exclusion criteria. Three patients refused to participate in the study after signing informed consent and allocation (2 from the rTMS-b group and 1 from the sham group). Five patients dropped out because of complications (3 from the rTMS-b group and 2 from the sham group). Finally, 45 participants completed the study (rTMS-w group, n=18; rTMS-b group, n=13; sham group, n=15) (Figure 1). All three groups were balanced at baseline with respect to the severity of aphasia, time since onset, participant age, gender and concomitant diseases (*P*>0.05) (Table 1).

### 3.2. Treatment Effects

**Figure 2:**
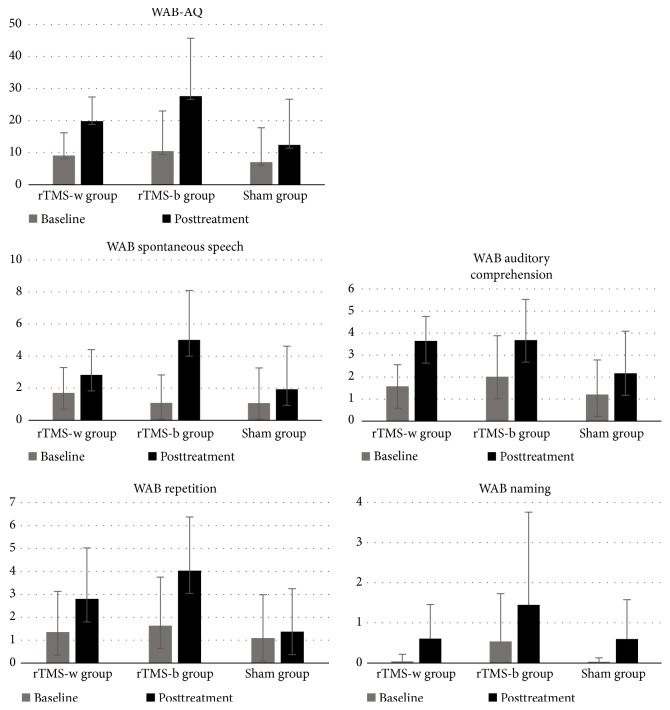
Language performance regarding pre- and posttreatment. Graphs showing means at baseline and after treatment for the three groups across WAB-AQ scores and WAB subtests.

A comparison of performance between the rTMS-w, rTMS-b and sham groups across baseline and posttreatment is shown in Figure 2 and Table 2. A series of two-way repeated measures analysis of variances (ANOVA) were conducted. Significant differences in the interactions between group and time were found on the following WAB-AQ and the WAB subtests: WAB-AQ scores (*P*<0.001), WAB spontaneous speech scores (*P*<0.001), WAB auditory comprehension scores (*P*=0.005) and WAB repetition scores (*P*<0.001). Post Hoc analyses were conducted between the three groups at baseline and posttreatment (repeated measured* t*-test). There was no significant difference in WAB scores for spontaneous speech, auditory comprehension, naming, repetition or AQ at baseline among the three groups (*P*>0.05). A significant change in auditory comprehension differences was found only between the rTMS-w group and the sham group (*P=*0.001, 95% CI (-1.73, -0.46)). The change in spontaneous speech remained significantly different between the rTMS-b group and the sham group (*P*≤0.001, 95% CI (-0.564, -0.235)). No significant difference was found for the changes in WAB scores for AQ (*P*=0.083, 95% CI (-0.413, 0.026)) and auditory comprehension (*P*=0.240, 95% CI (-0.270, 1.051)) between the rTMS-b group and the rTMS-w group.

## 4. Discussion

The present randomized sham-controlled study investigates the effect of inhibitory rTMS over the right pIFG or the right pSTG combined with SLT on language recovery from subacute global aphasia. A relatively short treatment period of rTMS combined with SLT promoted language recovery for rTMS over the right pIFG and pSTG as opposed to the sham group. LF-rTMS inhibited the right pSTG and led to significantly higher gains in auditory comprehension and repetition, whereas LF-rTMS inhibited the right pIFG and caused changes in spontaneous speech and repetition. These results provide evidence that rTMS may be an effective treatment tool for subacute stroke with global aphasia.

The present study suggested that the efficacy of rTMS on language recovery may be based on the stimulation sites. This study is the first to compare two stimulation sites in a randomized study. We chose global aphasia because these patients have difficulty involved in SLT due to their severely impaired auditory comprehension. Meanwhile, patients with global aphasia displayed little effect from SLT, whose therapeutic effects are quite variable and usually modest (Brady et al., 2012). Previous research has elucidated increased right hemisphere activation in individuals with left hemisphere lesions [21]. Such activation suggests that the brain may be employing a compensatory strategy which could potentially be maladaptive. Low-frequency rTMS provides an opportunity to normalize language function by inhibiting maladaptive brain activation and increasing adaptive activation. The underlying mechanisms for the LF-rTMS over the right two stimulations in patients with global aphasia were needed further study.

The results suggested that LF-rTMS inhibited the right pSTG and significantly improved language performance in WAB-AQ scores, auditory comprehension, and repetition. These results suggest that the related areas on the right hemisphere may contribute to recovery from aphasia when stimulated. These findings also corroborate a small pilot study by Abo et al. [13], who reported that low-frequency rTMS improved language function over the STG of the temporal lobe in patients with fluent aphasia. We also found that the application of LF-rTMS to the right pIFG displayed apparently beneficial changes in WAB-AQ, spontaneous speech, and repetition. This result is consistent with previous studies that showed that LF-rTMS over the right prIFG exhibited superior performance in spontaneous speech, comprehension, repetition, naming, and AQ in nonfluent aphasia [3, 8, 22, 23]. However, in our study, only small changes were observed in the recovery of naming ability between pre- and posttreatment with rTMS for global aphasia. The reason may be that there is a severe degree of speech injury in poststroke patients with global aphasia. The therapeutic effect of LF-rTMS over the right pSTG or prIFG may be mediated by increased inhibition of the contra-lesional hemisphere, thus restoring balance that enables the undamaged parts of the language area to function properly. The left anterior temporal lobe is not included in the MCA territory [24]. Furthermore, recovery of auditory comprehension in aphasia after left MCA infarction depends on the reorganization of the remaining language cortex of the left anterior temporal lobe [25]. These studies may provide a theoretical basis for the recovery of auditory comprehension in aphasic patients.

### 4.1. Study Limitations

The present study also has some limitations. Long-term effects have not been observed, and we did not have functional MRI available to investigate the change in activation of the language cortex before and after rTMS treatment.

## 5. Conclusions

Many studies have reported that low-frequency rTMS is beneficial for rehabilitating patients with aphasia, but the ideal stimulation sites for rTMS are not known. Low-frequency rTMS applied to the right pIFG and pSTG can be assumed to be an effective treatment for global aphasia following subacute stroke. Even immediately after the 15-day treatment, LF-rTMS inhibited the right pSTG and promoted significantly increased gains in auditory comprehension and repetition, whereas LF-rTMS inhibited the right pIFG and apparently caused changes in spontaneous speech and repetition. Further investigations are necessary to explore the neural mechanisms that underlie the differences in functional recovery observed between the different stimulation sites in this study.

## Figures and Tables

**Table 1:** Summary of patients' characteristics.

	rTMS-W group (n=18)	rTMS-b group (n=13)	Sham group (n=15)	Statistics	*P*
Gender, M/F	12/6	7/6	9/6	*χ* ^*2*^=0.528	0.768
Mean age, years (SD)	65.95 (8.53)	62.46 (10.95)	63.60 (16.71)	*F*=-0.561	0.574
Time of onset, days (SD)	55.90 (19.41)	50.58 (23.80)	61.20 (22.66)	*F*=-0.917	0.407
Hypertension (n)	14	11	12	*χ* ^*2*^=0.528	0.768
Diabetes (n)	5	5	7	*χ* ^*2*^=0.152	0.697
Coronary artery disease (n)	2	3	3	*χ* ^*2*^=0.073	0.787
Atrial fibrillation (n)	4	3	3	*χ* ^*2*^=0.167	0.682
WAB-AQ scores (SD)	9.07 (7.12)	10.48 (12.49)	7.05 (10.67)	*F*=-0.427	0.655

**Table 2:** WAB-AQ and subtest performances of the rTMS-w group, rTMS-b group, and sham group at pre- and posttreatment.

Test	Baseline Pre-rTMS			Post rTMS			F	ANOVA Group ^*∗*^Time (P)
	rTMS-w (n=18)	rTMS-b (n=13)	Sham (n=15)	rTMS-w (n=18)	rTMS-b (n=13)	Sham (n=15)		
Spontaneous speech	1.70±1.59	1.08±1.75	1.07±2.19	2.83±1.58	5.00±3.08	1.93±2.69	17.512	0.001
Auditory comprehension	1.58±0.98	2.02±1.86	1.21±1.57	3.64±1.11	3.68±1.85	2.17±1.92	6.099	0.005
Repetition	1.35±1.78	1.63±2.12	1.09±1.89	2.80±2.22	4.03±2.34	1.37±1.87	9.331	0.001
Naming	0.04±0.18	0.54±1.19	0.03±0.10	0.61±0.85	1.45±2.31	0.60±0.98	0.428	0.655
AQ	9.06±7.12	10.48±12.49	7.05±10.67	19.79±7.53	27.59±18.06	12.37±14.26	11.977	0.001

## Data Availability

The data used to support the findings of this study are included within the article.

## Additional Points

*Suppliers. *(a) MagPro TMS, MagVenture Company, Farum, Denmark. (b) SPSS software version 22.0 for windows; IBM.
